# An Efficient Method for Dorsal Root Ganglia Neurons Purification with a One-Time Anti-Mitotic Reagent Treatment

**DOI:** 10.1371/journal.pone.0060558

**Published:** 2013-04-02

**Authors:** Rui Liu, Gou Lin, Hanpeng Xu

**Affiliations:** 1 The Geriatric Department, Tangdu Hospital, The Fourth Military Medical University, Xi'an, Shaanxi Province, PR China; 2 LONI, Department of Neurology, University of California Los Angeles, Los Angeles, California, United States of America; 3 The Basic Medical School, The Fourth Military Medical University, Xi'an, Shaanxi Province, PR China; IBMC - Institute for Molecular and Cell Biology, Portugal

## Abstract

**Background:**

The dorsal root ganglia (DRG) neuron is an invaluable tool in axon growth, growth factor regulation, myelin formation and myelin-relevant researches. The purification of DRG neurons is a key step in these studies. Traditionally, purified DRG neurons were obtained in two weeks after exposure to several rounds of anti-mitotic reagent.

**Methods and Results:**

In this report, a novel, simple and efficient method for DRG purification is presented. DRG cultures were treated once with a high-dose anti-mitotic reagent cocktail for 72 hours. Using this new method, DRG neurons were obtained with 99% purification within 1 week. We confirmed that the neurite growth and the viability of the purified DRG neurons have no difference from the DRG neurons purified by traditional method. Furthermore, P0 and MBP expression was observed in myelin by immunocytochemistry in the DRG/SC co-culture system. The formation of mature node of Ranvier in DRG-Schwann cell co-culture system was observed using anti-Nav 1.6 and anti-caspr antibody.

**Conclusion and Significance:**

The results indicate that this high dose single treatment did not compromise the capacity of DRG neurons for myelin formation in the DRG/SC co-culture system. In conclusion, a convenient approach for purifying DRG neurons was developed which is time-saving and high-efficiency.

## Introduction

The non-neuronal cells always made the study of neurons complicate *in vitro.* Dorsal root ganglia provide a unique source of readily identifiable neurons, easily distinguishable from non-neuronal cells [1]. A substantial body of evidence indicates that adult mammalian DRG neurons are able to survive and regenerate in culture [2], [3], [4]. For now, there are several works on dealing with purified populations of these neurons [5], [6], [7]. Cultures of purified DRG neurons are an invaluable tool for the study of axon growth, what's more, the DRG neurons could use in myelin formation. These studies required an in vitro survival bioassay using purified adult neuronal populations in order to avoid interference by non-neuronal cells.

Nearly three decades ago, researchers developed a dorsal root ganglia (DRG) neuron/SC co-culture system for myelin research *in vitro* [8], [9]. In this co-culture system model, purification of DRG neurons is mandatory. DRG cultures are routinely purified before SCs are added. This is because the over-expanding non-neuronal cells could interfere with myelin formation and interrupt results [10]. To obtain a pure DRG neuron population, anti-mitotic reagents, such as fluorodeoxyuridine, were introduced into the culture medium to eliminate non-neuronal cells [11], [12]. However, the cultures had to be treated for prolonged anti-mitotic exposure and carried through several rounds to finish, which is a time consuming, laborious and expensive procedure. It is not uncommon that after 2 weeks and more than 3 rounds of treatment, the purity of DRG neurons still do not reach the experimental requirements [13]. The prolonged purification procedure also increased the handling risk and the risk of additional contaminations, such as bacterial infection.

To overcome these drawbacks, a new method was developed to purify DRG neurons in culture more efficiently. In this innovative method, only one high-dose anti-mitotic reagent treatment was needed. DRG neurons could be readily purified to 99% within 1 week without compromising their capacity for neurite growth and myelin formation.

## Materials and Methods

### Animals

Sprague-Dawley rats were used in this experiment, with all animal protocols approved by the Animal Experiment and Care Committee of The Fourth Military Medical University, China.

### Isolation, purification and culture of DRGs

The ganglia were dissected out from embryonic Spargue-Dawley rats (15 days gestation) and were incubated in ice-cooled L15 medium (Invitrogen, Carlsbad, CA). The ganglia were then transferred into 35 mm culture dishes containing 2 mL trypsin/EDTA (0.125%/0.05%, Sigma-Aldich, Saint Louis, MO) and incubated at 37 °C for 15 min. After digestion, ganglia were transferred to a 15 mL conical centrifuge tube with 5 mL of L15 medium containing 10% heat-inactivated fetal bovine serum (FBS, Hyclone Labs., Logan, UT) and DNase (10 µg/mL Sigma-Aldich, Saint Louis, MO). Ganglia were triturated with a fire-polished Pasteur pipette (Fisher Scientific, Pittsburgh, Pa) until the suspension was homogeneous. The cell suspensions were filtered through a cell-strainer (40 µM, BD Biosciences, Bedford, MA) and centrifuged at 250 g for 10 min. DRG pellets were washed one more time with NG medium (neurobasal medium, 2% B27 supplement, 2 mM l-glutamine, 0.4% glucose and 50 ng/ml 2.5 S NGF, Invitrogen, Carlsbad, CA) and resuspended in NG medium.

Before DRG neuron culture, 12 mm coverslips (Fisher Scientific, Pittsburgh, Pa) were coated with polylysine and laminin (Sigma-Aldich, Saint Louis, MO) under sterile condition and then placed into 4-well plates (Nunc, Naperville, IL). 100 µL of DRG neuron suspension (approximately 5000–10000 cells) was added onto each coverslip in the 4-well plate. Cultures were incubated at 37 °C, with 5% CO_2_. 24 h later, 150 µL of NGF stock solution (40 µM of 5-fluorodeoxyuridine and uridine, 1 µM Arabinofuranosyl Cytidine (Ara C, Sigma-Aldich, Saint Louis, MO) in NG medium) was added into each well to make the final concentration of 5-fluorodeoxyuridine/uridine 20 µM. Cultures were incubated at 37 °C in a 5% CO_2_ incubator. After 72 hours, 2/3 of the NGF stock solution was changed to NG medium and the cultures were re-fed every 2 days with NG medium. After three medium changes, the neurons were ready for the Schwann cell addition. The purification processes were observed and recorded under phase contrast microscope, and some cultures were fixed and subjected to immunocytochemistry staining for non-neuronal cell detection.

### Isolation, purification and culture of SCs

SCs were prepared from the sciatic nerves of neonatal rat pups (P2-P6) by the method described previously [14]. Briefly, SCs were purified using the Thy1-complement method and were expanded in D media (DME, 10% FBS, and 2 mM l-glutamine) supplemented with 4 µM of forskolin and 5 ng/mL of the EGF domain of rhNRG-1-β1 (R&D Systems, Minneapolis, MN) over a period of 2–3 weeks. SCs were then maintained in D media for 3 days before use. In addition, rat SCs were expanded *in vitro* and kept in liquid nitrogen for later use. All of the SCs used in myelin formation experiments were not passaged more than 4 times.

### DRG/SC co-cultures

SCs were digested and washed once with DMEM (Invitrogen, Carlsbad, CA) containing 10% FBS, and then resuspended in C medium (MEM, 10% FBS, 2mM l-glutamine, 0.4% glucose, and 50 ng/mL 2.5 S NGF). 100 µL (approximately 200,000 cells) of SCs was added to each of the DRG cultures in C media. After 3 days, the co-cultures were supplemented with 50 µg/mL of ascorbic acid to initiate basal lamina formation and myelination. Myelination was allowed to proceed for up to 21 days. The process of myelination was observed and recorded under phase contrast microscope.

### Cell viability assay

The cell viability was determined by Trypan Blue exclusion assay as described previously with minor modifications [15]. In briefly, Cell counts were performed 7 d after plating to determine whether cells were being lost or becoming nonviable during incubation period. Total cell numbers per well were determined. On the day of assay, cells cultured with NG medium were gently washed with warmed F-12 media and then incubated for 5 min in a 0.4% solution of Trypan Blue (Sigma). The cells were subsequently washed three times with F-12 before counting under a light microscope. Cells demonstrating dye uptake were classed as nonviable.

### Measurement of Neurite lengths

Neurite growth (length of longest neurite) were determined using phase contrast microscopy and computer-based quantitative image analysis [16]. In briefly, the neurons cultured with NG medium were incubated at 37 °C, with 5% CO_2_ for 7 d. Cell were fed with NGF on days 0, 2, 4, 6. Neurite growth was determined by manually tracing the length of the longest neurite per cell (using NIH Image software) for all cells in a field that had an identifiable neurite and for which the entire neurite arbor could be visualized. Data from the two fields in each well were pooled, and each well was designated as an “n” of one. Experiments were repeated at least three times using cultures prepared on separate days.

### Immunocytochemistry

The DRG cultures were washed with 0.1 M PBS and fixed in 4% paraformaldehyde (PFA) for 10 min, followed by cold methanol for 20 min. The cells were washed 3 times with PBS after each fixation step. Non-specific antibody-binding sites were blocked with block medium (10% FBS in PBS) for 30 min at room temperature and washed again with PBS. The purified DRG culture coverslips were then incubated at room temperature with a mouse anti-p75NTR monoclonal antibody (dilution 1:500), a mouse anti-S100 monoclonal antibody (dilution 1:100), and a mouse anti-fibronectin polyclonal antibody (dilution 1:100), which were all obtained from Abcam (Cambridge, MA). The corresponding Alexa488-conjugated secondary antibodies (Invitrogen, Carlsbad, CA) and DAPI (Invitrogen, Carlsbad, CA) were added to visualize the cells. All positive cells in each coverslip were counted and their percentage in the total number of cells was calculated.

To observed the formation of the myelin, the DRG neuron/SC co-cultures (14 days after initiation myelin formation) were then labeled with primary antibodies, which included mouse anti-myelin basic protein (MBP) monoclonal antibody (dilution 1:1000, Abcam, Cambridge, MA), rabbit anti-myelin protein zero (P0) monoclonal antibody (dilution 1:1000, Abcam, Cambridge, MA), mouse anti-caspr monoclonal antibody (dilution 1:200, NeuroMab, Davis, CA), rabbit anti-Nav1.6 monoclonal antibody (dilution 1:200, Abcam, Cambridge, MA). After three washes with PBS, the coverslips were further incubated with Alexa Fluor 488 mouse anti-rabbit IgG and Alexa Fluor 594 goat anti-mouse IgG (Invitrogen, Carlsbad, CA) for 60 min at room temperature. After a final wash in PBS, the slides were mounted with mounting fluid (DAKO Ltd., Carpenteria, CA) and visualized under a fluorescence microscope (Olympus, Tokyo, Japan). The images were digitally recorded and processed with Image-Pro Plus (Media Cybernetics, Atlanta, GA).

### Statistical analysis

One-way analysis of variance (ANOVA) was used to compare values among multiple groups followed by Dunnett's test. Differences were considered statistically significant at P<0.05. All experiments were repeated at least three times.

## Results

### Characterization of Dorsal Root Ganglion Cell Cultures

**Figure 1:**
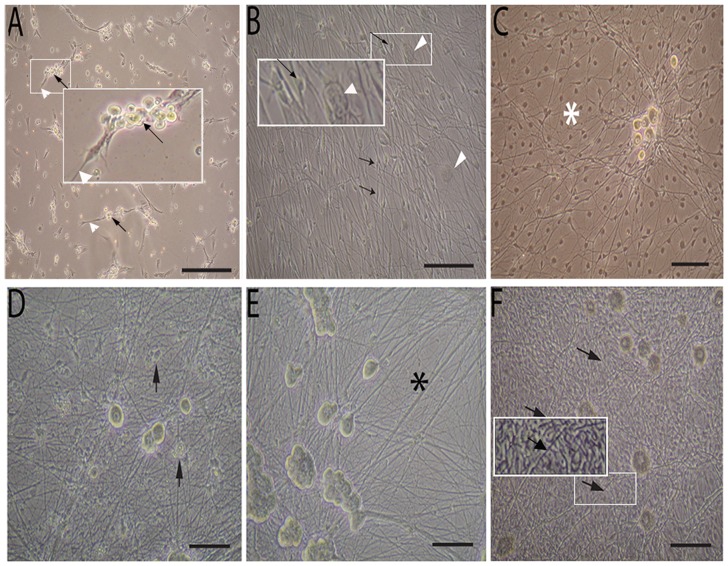
Phase contrast images of DRG purification and myelination. (A) DRG cultures at 24 h before adding antimitotic reagents cocktail, black arrow show DRG neurons, white arrowhead show non-neurons cells; (B) DRG cultures 72 h without high dose antimitotic reagent cocktail treatment, Schwann cell associated with neuritis (arrow) and other big flat non-neuron cells migrated out and grew between neuritis (arrowhead); (C) DRG cultures at 7 day without cocktail treatment, Schwann cell and other non-neuron cell formed a cellular lawn (white star) with DRG neurons sitting on them; (D) DRG cultures 72 h after cocktail treatment, abundant neurites and neurons were observed, dead non-neuron cell debris (black arrow) floating in the medium; (E) DRG cultures at 7 day after cocktail treatment, a pure DRG population without dead non-neuron cells established, single or small cluster of DRG neurons showed typical morphologies with no signs of damage, clean neurites without non-neuron cells (black star); (F) Cocktail treated DRG cultures 14 days after seeding Schwann cells and initiating myelination with ascorbic acid, abundant mature myelin had formed (arrow). Scale bar 50 µm.

In the dissociated DRG culture, both DRG neurons and non-neuronal cells were attached on the substrate within 2 hours after seeding. Under phase contrast microscopy, these two cell populations could be easily identified according to their morphologies. DRG neurons were characterized by their unique bright round cell bodies, clear nuclei, and sometimes, nucleoli were also observed (Fig. 1 A). During the first 24 hours, DRG neurons grew out of neurites and extended into the surrounding spare area. These neurons also formed small clusters. Two major types of non-neuronal cells were identified: the SCs were spindle shaped neurites associated with small-size cells, and the fibroblasts were dark, flat shaped large cells which did not associate with neurites (Fig. 1 B).

When these cultures were not treated by anti-mitotic reagents, the neurites kept on growing and the DRG neurons became larger with time. This was accompanied by the rapid proliferation and migration of non-neuronal cells. After 7 days, the non-neuronal cells become confluent and formed a cell lawn with DRG neurons and neurites sitting on top (Fig. 1 C). Usually, there was a slight cell crisis period between 2 to 3 days after seeding, but this did not change the gross cellular growth.

When high dose anti-mitotic reagent cocktails (20 µM of Fudr/urine plus 0.5 µM of Ara C) were added to the DRG culture after 24 h cell seeding, no obvious cell death was found in the following 24 h, and the non-neuronal cells continued to proliferate and migrate as in the non-treated cultures. Large-scale cell death occurred after 24 h, and this cellular death wave continued and climaxed at 72 h. Most of the cellular death occurred in non-neuronal cells, with no significant influence on the DRG neuron morphology and neurite growth observed. At 72 h, dead cells and cellular debris were floating in the medium and a relatively pure DRG neuron population was obtained with some residual non-neuronal cells. After the medium was changed twice, dead cells were cleaned out and, at 7 days, a pure neuron population was obtained with few non-neuronal cells (Fig. 1 D). In the preliminary experiment, when a higher dose (more than 20 µM) of anti-mitotic reagent was added, both neurons and no-neuronal cells were killed, while a lower dose did not eliminate all of the non-neuronal cells. It was found that serum-containing medium also decreased the non-neuronal cell sensitivity to anti-mitotic reagents (data not shown).

The purity of the neuron population was evaluated by phase contrast imaging and cell type-specific antibodies. In the 7 day cultures, phase contrast microscopy showed that the non-neuronal cells were less than 1% of all of the cultures, with more than 99% of cells being comprised of neurons (Fig. 1 E). Moreover, cocktail treated DRG cultures 14 days after seeding Schwann cells, the myelin neurites were observed under phase contrast microscope (Fig. 1 F).

**Figure 2:**
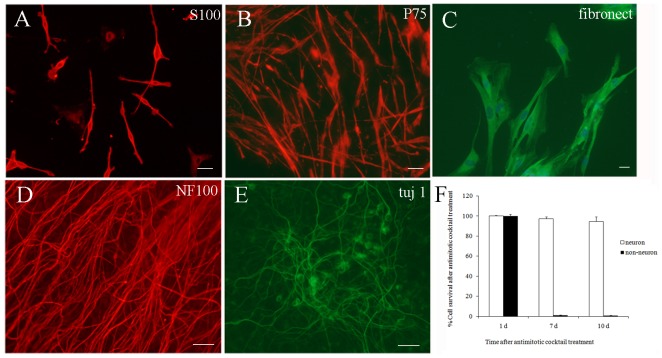
The survival of DRG neurons and non-neuronal cells after cocktail treatment. (A–F) The immunocytochemistry for DRG neurons and non-neuron cells (on day 4). A–C: non-neuron cells (include all the S100^+^, P75 NRT^+^ and fibronect^+^ cells); Scale bar 10 µm; D–E: DRG neurons (include the NF-100^+^ and tuj 1^+^ cells); Scale bar 50 µm. F. The survival of DRG neurons and non-neuronal cells after cocktail treatment. Cell survival assessed as mean cells counts per well after 1, 7, 10 days culture. Data are means ± SEM. (n = 3). (Dunnett's test; P>0.05).

To further confirm the purity of the culture, some cultures were changed back to serum-containing medium, which facilitated the re-growth of non-neuronal cells. In the 10 day cultures, the results showed no significant non-neuronal cell growth under fluorescence microscope. Immunocytochemistry results showed that there has few non-neuron cells, such as S100 (+), P75 NRT (+) and fibronect (+) cells. The proportion of non-neuronal cells was less than 1% (Fig. 2).

### Cell Viability

**Figure 3:**
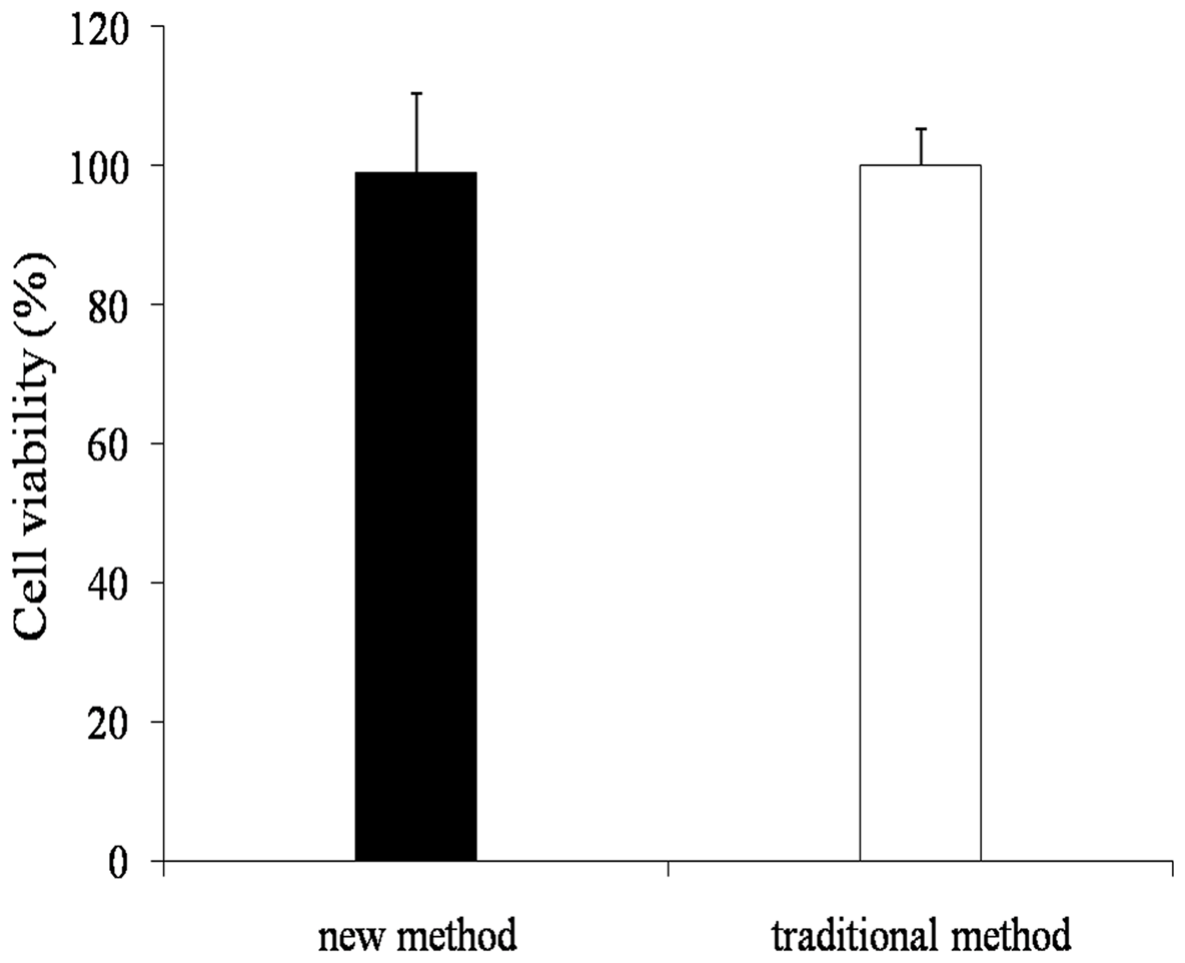
DRG neurons viability (compared with traditional method). Data are means ± SEM. (n = 5). No significantly difference was observed in cell viability between new method and traditional method. (Dunnett's test; P>0.05). At the end of 7 d culture period the vast majority of DRG neurons also remained viable, as evidenced by the high level of Trypan blue exclusion in all culture conditions.

To determine whether the high dose single treatment induced a more general cytotoxicity in DRG cells, cell viability was examined at 7 days after DRG neurons purification (Fig. 3). The results showed that high dose single treatment did not affect cell viability. There is no difference between the new method and the traditional method.

### Neurite growth in DRG cultures

**Figure 4:**
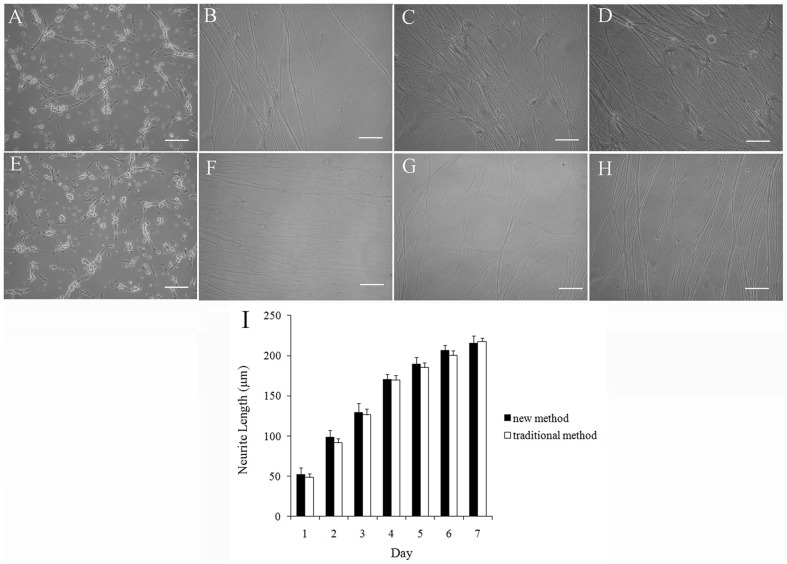
Neurite growth of purified DRG neurons. Cells were fed with NGF on day 0, 2, 4, 6. (A–H) The representative phase contrast images of DRG neurons. A–D: Neurite growth of DRG neurons which purified by traditional method on day 1 (A), 3 (B), 5 (C), 7 (D). E–H: Neurite growth of DRG neurons which purified by new method on day 1 (E), 3 (F), 5 (G), 7 (H). I: The comparison of neurite growth of purified DRG neurons between traditional method and new method. Data are means ± SEM. (n = 9). No significantly difference was observed in neurite length between new method and traditional method. (Dunnett's test; P>0.05); Scale bar 50 µm.

A neurite refers to any projection from the cell body of a neuron, such as axon and dendrite. Neurite is a very important symbol when speaking of immature or developing neurons, especially of cells in culture. Therefore, we have examined the components of neurite outgrowth in cultures of adult rat DRG neurons (Fig. 4). The results showed that neurite length increased in a time-dependent process. There is no significant difference between our present method and the traditional method.

### The myelin formation of purified DRG culture

**Figure 5:**
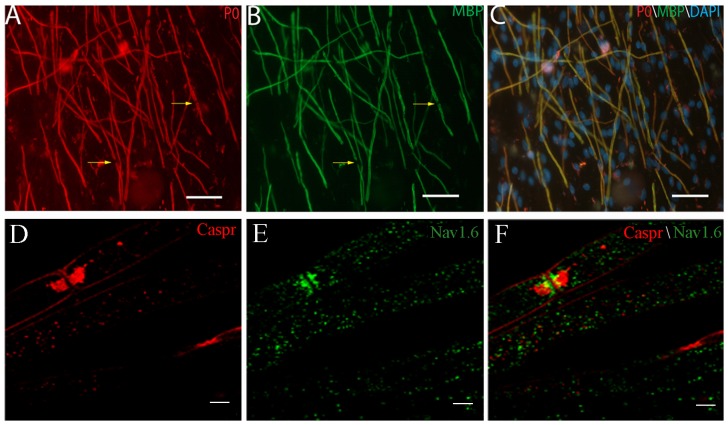
Myelin formation detected by immunofluorescence for myelin specific markers. (A) Peripheral protein zero (P0, red), (B) Myelin basic protein (MBP, Green) and (C) Merge of the A and B and count staining with DAPI (blue) for nucleus. Abundant mature myelin and Ranvier node (arrow) were observed; Scale bar 50 µm. (D) Caspr (red), (E) Nav1.6 (green) and (F) Merge of the D and E. The formation of mature node of Ranvier in DRG-Schwann cell co-culture using one time high dose impact treatment were observed; Scale bar 5 µm.

Although the viability and morphology of purified DRG neurons did not show any difference after the single high dose anti-mitotic reagent treatment, to those purified by traditional multiple treatments, it is unclear whether the neuronal capacities were altered. Myelin is a specialized membrane component which plays a critical role in normal nervous system development and function. It is necessary for the salutatory conduction of action potentials to propagate more efficiently and more rapidly [17]. DRG neuron/SC co-culture system is a powerful technique which could initiate myelinogenesis myelination. Therefore, to further confirm the purified DRG neurons did not show any difference from traditional method, purified SCs were added to the purified DRG neurons and their myelination was initiated according to the commonly used method. The myelin formation process followed the same time course as the traditional method. After Schwann cell addition, the cells readily associated and aligned with neurites. Ascorbic acid in the medium initiated the myelin formation at around 7 days. Myelin neurites were observed at day 14 and lasted for 4 weeks without any difference from those of the traditional method in morphology. Myelin specific P0 and MBP staining showed typical myelin fragments and Ranvier nodes in this co-culture system (Fig. 5). Nodes of Raniver were defined by staining with anti-Nav 1.6 and anti-caspr antibody. Staining with anti-Nav1.6 antibody is shown in green and staining with anti-caspr antibody is shown in red. Immunofluorescent labeling of DRG/SC co-culture with anti-Nav1.6 antibody showed intense labeling of nodes of Ranvier that were identified by (i) their morphology and (ii) colabeling with antibodies against caspr [18]. The Caspr and Nav1.6 were staining showed the formation of mature node of Ranvier in DRG-Schwann cell co-culture system using one time high dose impact treatment (Fig. 5).

## Discussion

As an invaluable research model, purified DRG neurons had been broadly used in various studies, including axon growth and regeneration, degeneration, apoptosis, growth factor regulation, ion channels, myelin formation and demyelination disease [19], [20]. Cultures of purified adult neurons are an invaluable tool for the study of the trophic factors required for the survival and regeneration of the adult nervous system[7]. In this culture system, it is a pre-requisite to eliminate the contamination of non-neuronal cells which can make experimental manipulation difficult [10]. Currently, to obtain pure DRG neurons, dissociated DRG neurons are usually treated by anti-mitotic reagents for three or more times, in either serum-containing medium or serum-free medium [21], [22]. However, this multi-round treatment process usually requires 2 weeks. In myelin culture relevant studies, it usually took 6–8 weeks to get an ideal myelin culture for further assays, which was a time-consuming, laborious and expensive process.

**Figure 6:**
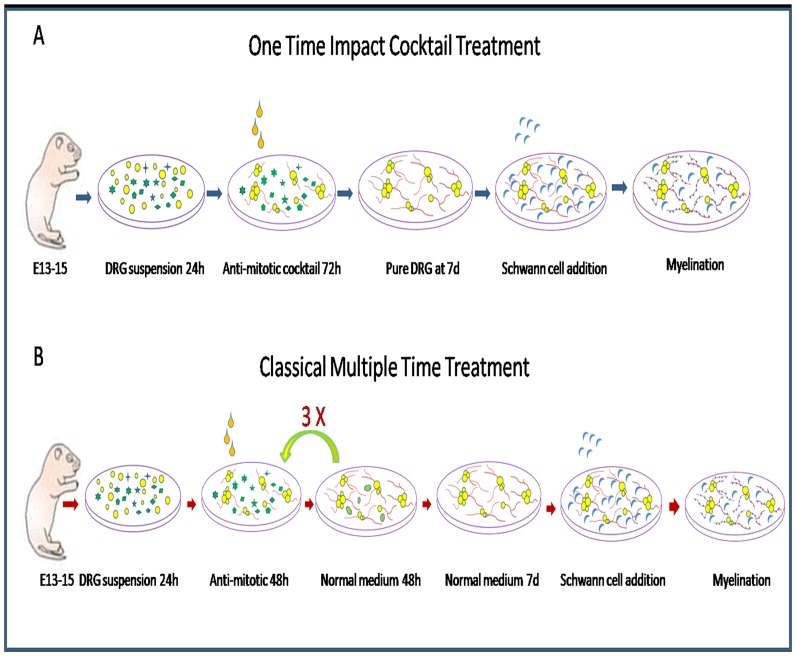
Schematic diagram of the experimental procedure of the new method comparison with tradition method. (A) The new one time high dose impact treatment method, after 24 h in NG medium, DRG cultures were treated with NGF stock solution containing 20 µM FUDR plus 0.5 µM Ara C cocktail for 72 h, then switched to normal NG medium, at 7 day, a pure DRG neuron population (>99%) were obtained. At 10 day, these DRG neurons were ready for SC addition. (B) The classical multiple time treatment method, after 24 h in normal medium, DRG cultures were treated with NGF stock solution containing 10 µM FUDR for 48 h, then switched to NG medium for 48 h, this cycle was repeated at least 3 times; then DRG culture were maintained in NG medium for 1 week with 3 times medium changes to obtain the pure DRG population and these cultures were ready for Schwann cell addition.

In this report, a more efficient purification method has been presented that yields highly-enriched DRG neurons within a short time. The DRG neuronal purification procedure is shown in Fig. 6. This method is different from traditional multi-round anti-mitotic treatments, as the DRG neuron culture is treated with a single high-dose (20 µM instead of 10 µM) anti-mitotic cocktail. This also involves a prolonged treatment time (72 hours instead of 48 hours). After this treatment, non-neuronal cells in DRG neuron cultures were depleted completely and pure DRG neurons were obtained within 1 week. This highly-efficient and simple method made DRG neuron purification very easy to perform and could be completed within a short time. Moreover, the neurite growth and the capacity of myelin formation of purification of DRG neurons were not compromised by high-dose anti-mitotic treatments.

Using this method, it was possible to obtain high-purity DRG neurons consistently in large amounts within a short time. The high purity DRG neurons could utilize in the studies of neurobiology, such as axon growth, neuron and glial cell interaction and gene function studies [23], [24]. Obtained high purified neurons made this method a good choice for the studies of neurobiology. The purified neurons were recently successfully used in a co-culture model to study the influence of astrocyte heterogeneity on axon growth [23].

To summarize, a simple and efficient method for DRG neuron purification was developed. Using this new method, high-purity DRG neurons were obtained within a short time. The purified DRG neurons had no differences in morphology and function from those purified by the traditional method. Thus, this research affords a basis for further studies by providing a simple and efficient method for the purification of DRG neurons.
